# One-step synthesis of hydrophobic clinoptilolite modified by silanization for the degradation of crystal violet dye in aqueous solution†

**DOI:** 10.1039/d0ra03151h

**Published:** 2020-06-15

**Authors:** Jian Jiao, Jihong Sun, Raza Ullah, Shiyang Bai, Chengwei Zhai

**Affiliations:** Beijing Key Laboratory for Green Catalysis and Separation, Department of Chemical Engineering, Beijing University of Technology Beijing 100124 China jhsun@bjut.edu.cn

## Abstract

Hydrophobic clinoptilolite (CP) was successfully synthesized *via* a silanization method using methyltriethoxysilane (MTS) or diethoxydimethylsilane (DMTS) as silane coupling agents. The structural and textural properties of the resultant hydrophobic CP were characterized using various methods. The effect of the amount of MTS or DMTS additive on the induction (nucleation) and growth of CP were also investigated, and the apparent activation energy values for induction and growth periods were calculated, suggesting that the induction period is kinetically controlled, while the rapid growth process is thermodynamically controlled. Meanwhile, DMTS modification enhanced the hydrophobicity of CP compared with its MTS-modified counterpart and pure CP. Finally, various ZnO-supported CPs were used as photocatalysts for the removal of crystal violet from aqueous solution, demonstrating that ZnO/hydrophobic CP has the largest adsorption capacity and best removal performance. These results suggest that hydrophobic CP, as an adsorbent or support, has the most potential for applications in separation and catalysis.

## Abstract 2

One-step synthesis of hydrophobic CPs was demonstrated, in which the kinetically-controlled induction period and thermodynamically-based rapid growth process were elucidated.

## Graphical abstract

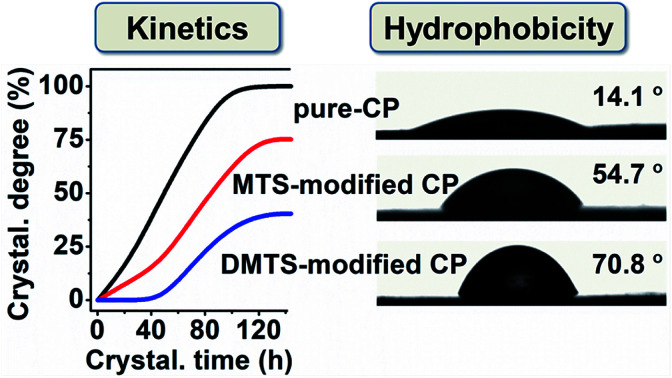

## Introduction

As a typical non-biodegradable dye, crystal violet (C_25_H_30_N_3_Cl, abbreviated as CV, with a solubility in water of 50 g L^−1^ at 25 °C and maximum adsorption wavelength with a characteristic peak of 580–583 nm) is presently largely used in industry, but may cause skin irritation, and even cancer, in humans and other animals.^1^ Therefore, it is highly desirable to remove CV dye from industrial wastewater before it is discharged into streams and rivers. Recently, heterogeneous photocatalytic degradation has become an economical advanced oxidation technique/process (AOT/AOP) and a promising technology for wastewater treatment, because it is environmentally friendly and has been applied in the removal of dyes from wastewater.^2^ ZnO semiconductors have several favorable properties,^3^ including that they exhibit a 3.37 eV direct band gap, have low production costs and a massive binding energy of 60 meV, and therefore have been found to be one of the most promising and potential photocatalysts for the photocatalytic degradation of various organic pollutants.

Presently, clinoptilolite (CP) has attracted extensive attention in various applications in the fields of environmental catalysis, drug delivery, and gas separation.^4–6^ However, due to its harsh synthesis conditions and the presence of miscellaneous crystals in the product once synthesized, a number of researchers have focused on synthesis methods that require mild conditions. An early report from Satokawa and Itabashi^7^ detailed using a “seed” method to synthesize CP, then, Chi and Sand^8^ systemically demonstrated the synthesis parameters with a large amount of additive seeds (1–10 wt%) at 120–200 °C for 1–7 days. Recently, Sun *et al.*^9,10^ synthesized highly-pure CP using aluminosilicate-containing sol as a structure guiding agent at 150 °C for 3 days.

Although CP has been preliminarily applied as a sorbent in solid-phase extractions for the enrichment or removal of trace amounts of heavy metals,^11^ gas separation,^12^ catalysis,^13^ drug release,^14^ and soil improvement,^15^ its poor hydrophobicity and therefore strong water adsorption ability strongly limit its performance under wet conditions. However, the hydrophobic modification of synthetic CP is rarely reported because of its strict synthesis conditions. For these reasons, many studies have been explored on the surface hydrophobicity of natural CP. Accordingly, an early synthesis of hydrophobic CP by Allen *et al.*^16^ was carried out *via* decationisation treatment with NH_4_Cl solution and then dealuminisation with HCl solution. As reported by Bel'chinskaya *et al.*,^17^ natural CP can be modified by hydrophobization with organosiloxanes, and therefore its adsorption selectivity for toluene and water from the gas phase can be easily adjusted by hydrophobization with surfactant octadecylammonium and alginate biopolymers. Wang *et al.*^18^ further elucidated the surface modification of natural CP *via* the exchange of hydrophilic inorganic cations with hydrophobic organic ions. Similarly, Zhang *et al.*^19^ demonstrated the effects of functionalized silanol with tetraalkylammonium on the enhancement of the hydrophobicity of NaY zeolites. Kawai and Tsutsumi^20^ prepared hydrophobic Na-type faujasite *via* SiCl_4_ treatment during dealumination-silicon exchange procedures, which was very useful in improving its adsorption of sodium dodecylsulfate from aqueous solution.

However, few reports are available to help guide the conformation and role of the silane coupling agent during the synthesis procedure of CP, which is very important for understanding the modified behaviour and predicting its environmental applications in wet systems, such as for dye removal or contaminant adsorption. This report describes the one-step synthesis of hydrophobic CP modified using the silane coupling agents methyltriethoxysilane (MTS) and diethoxydimethylsilane (DMTS). The experimental parameters were systematically investigated in depth so as to obtain the optimal synthesis conditions over a narrow crystallization field, such as crystallization time and temperature, as well as additive amounts of the silane coupling agent. In particular, the possible effects of the mechanism of the silane coupling agent on the structural properties of the resultant hydrophobic CP are proposed after using various characterization methods, such as X-ray diffraction (XRD), N_2_ adsorption–desorption isotherms, scanning electron microscopy (SEM), transmission electron microscopy (TEM), Fourier-transform infrared (FT-IR) spectroscopy, UV-Raman spectroscopy, the temperature-programmed desorption of ammonia (NH_3_-TPD), ^29^Si nuclear magnetic resonance (NMR) spectroscopy, thermogravimetric (TG)-differential scanning calorimetry (DSC), and water contact angle (WCA) measurements. Finally, zinc chloride was used as a Zn source to be loaded onto the modified hydrophobic CPs, as a photocatalyst, their degradation performance against crystal violet (CV) dye in aqueous solution was preliminary investigated and compared with those of natural CP and synthesized pure CP as supports.

## Experimental

### Materials

Sodium hydroxide (NaOH, analytical grade (AR), 96.0% purity) and potassium hydroxide (KOH, AR, 82.0%) were purchased from Beijing Chemicals Works. Aluminium hydroxide (Al(OH)_3_, AR, 99.5%) and zinc chloride (ZnCl_2_, AR, 98.0%) were supplied from Tianjing Fuchen Chemicals Works. Silica sol (30 wt%) was obtained from Qingdao Ocean Chemical Co., Ltd. MTS (AR, 99.0%) and DMTS (AR, 99.0%) were provided by Nanjing Capatue Chemical Co., Ltd. CV dye (high purity biological stain) was obtained from J & K Co., Ltd. Deionized water with a resistivity of 18.25 MΩ cm was used in all experiments.

### Synthesis of hydrophobic CP

Natural CP was provided by Pure Mulan Co., Ltd. It was firstly calcinated at 450 °C for 6 h, and then fully ground using a 100 mesh sieve screen. After that, the obtained white powder was used as seeds, which belonged to the heulandite (HEU) phase with lesser quantities of clays, quartz and amorphous, as characterized in the XRD pattern of Fig. S1 of the ESI† section.

Hydrophobic CP was synthesized *via* a hydrothermal method with a molar ratio of Na_2_O : K_2_O : SiO_2_ : Al_2_O_3_ : H_2_O = 1.38 : 1.38 : 11.18 : 1 : 294.30. The detailed process was as follows: 0.5192 g of NaOH, 0.7268 g of KOH, 0.7362 g of Al(OH)_3_, and 25 mL of water were weighed and then magnetically stirred for 3 h at 130 °C to obtain a clear solution as an aluminium source. 0–0.8 mL of silane coupling agent (MTS or DMTS) and 8.0–8.8 mL of silica sol were mixed as a silicon source. After that, the prepared silicon source was added dropwise into the aluminium source, meanwhile, 6–10 wt% of the abovementioned natural CP as a seed was added to it with stirring at room temperature. After 2 h, the homogenous solution changed into a white gel and was then crystallized at 110–170 °C in an oven for 1–7 days in a stainless steel autoclave fitted with a Teflon-lined container. Then, the autoclave was removed from the oven and allowed to cool to room temperature, the resultant solid was recovered by filtration on a Buchner funnel, washed with 500 mL of deionized water, and then dried in an oven at 100 °C overnight. The obtained products were referred to as CP-*z-x*M(or D)*y*, where *z* and *x* denote the crystallization temperature (°C) and the additive amount (mL) of the silicon coupling agent, M (or D) and *y* indicate the MTS (or DMTS) and crystallization time (h). More details on the starting composition and corresponding synthesized conditions are listed in Table 1. As shown in Table 1, CP-150-3 or CP-150-5 were used as the synthetic pure CP.

**Table 1:** Gel composition and corresponding synthesis conditions for CP

Sample code	Molar ratio of MTS (or DMTS) to total Si		Crystallization conditions	Product phase
	MTS	DMTS		
CP-150-5	0	0	150 °C, 5 d, 6 wt% seed	CP
CP-150-0.2M5	1.89	0	150 °C, 5 d, 6 wt% seed	CP + phillipsite
CP-150-0.4M5	3.79	0	150 °C, 5 d, 6 wt% seed	CP + phillipsite
CP-150-0.6M5	5.67	0	150 °C, 5 d, 6 wt% seed	CP + phillipsite
CP-150-0.8M5	7.56	0	150 °C, 5 d, 6 wt% seed	Phillipsite + CP
CP-150-3	0	0	150 °C, 3 d, 10 wt% seed	CP
CP-150-0.2D3	0	2.28	150 °C, 3 d, 10 wt% seed	CP
CP-150-0.4D3	0	4.56	150 °C, 3 d, 10 wt% seed	CP + amorphous
CP-150-0.6D3	0	6.84	150 °C, 3 d, 10 wt% seed	CP + amorphous
CP-150-0.8D3	0	9.11	150 °C, 3 d, 10 wt% seed	Amorphous + CP
CP-150-0.4M1	3.79	0	150 °C, 1 d, 6 wt% seed	Amorphous
CP-150-0.4M2	3.79	0	150 °C, 2 d, 6 wt% seed	Amorphous + CP
CP-150-0.4M3	3.79	0	150 °C, 3 d, 6 wt% seed	CP + phillipsite
CP-150-0.4M4	3.79	0	150 °C, 4 d, 6 wt% seed	CP + phillipsite
CP-150-0.4M6	3.79	0	150 °C, 6 d, 6 wt% seed	CP + phillipsite
CP-150-0.4M7	3.79	0	150 °C, 7 d, 6 wt% seed	Phillipsite + mordenite
CP-130-0.4M5	3.79	0	130 °C, 5 d, 6 wt% seed	Amorphous
CP-140-0.4M5	3.79	0	140 °C, 5 d, 6 wt% seed	Amorphous + CP
CP-160-0.4M5	3.79	0	160 °C, 5 d, 6 wt% seed	Phillipsite + CP + mordenite
CP-170-0.4M5	3.79	0	170 °C, 5 d, 6 wt% seed	Phillipsite + mordenite + CP
CP-150-0.6D1	0	6.84	150 °C, 1 d, 10 wt% seed	Amorphous
CP-150-0.6D2	0	6.84	150 °C, 2 d, 10 wt% seed	Amorphous + CP
CP-150-0.6D4	0	6.84	150 °C, 4 d, 10 wt% seed	CP
CP-150-0.6D5	0	6.84	150 °C, 5 d, 10 wt% seed	CP + phillipsite
CP-150-0.6D6	0	6.84	150 °C, 6 d, 10 wt% seed	CP + phillipsite + mordenite
CP-150-0.6D7	0	6.84	150 °C, 7 d, 10 wt% seed	CP + phillipsite + mordenite
CP-130-0.6D3	0	6.84	130 °C, 3 d, 10 wt% seed	Amorphous
CP-140-0.6D3	0	6.84	140 °C, 3 d, 10 wt% seed	Amorphous
CP-160-0.6D3	0	6.84	160 °C, 3 d, 10 wt% seed	CP + mordenite
CP-170-0.6D3	0	6.84	170 °C, 3 d, 10 wt% seed	Mordenite + CP + phillipsite

### ZnO/CP preparation

100 mL of ethanol was mixed with 20 mL of a 1 M KOH solution in a glass beaker under continuous stirring for 10 min. Then, 25 mL of an aqueous solution of 0.4 M ZnCl_2_ was added dropwise into the above solution under constant stirring for 0.5 h, and then transferred into different a Teflon-lined vessels along with 1 g of each of the natural CP, synthetic pure CP and hydrophobic CP. After that, the vessels were enclosed in stainless steel autoclaves and heated hydrothermally at 125 °C for 5 h. Finally, the products were filtered, washed thoroughly with deionized water and dried at 100 °C overnight to obtain the ZnO/natural CP, ZnO/synthetic pure CP, and ZnO/hydrophobic CP photocatalysts. For comparison, a 0.8 M ZnCl_2_ solution was also treated with natural CP and subjected to the same process as mentioned above.

### Characterization

The structures of the resultant CP and catalysts were determined from their XRD patterns (Beijing Purkinje General Instrument Co. Ltd) using a diffractometer equipped with a CuKα radiation source in the 2*θ* range of 5–75° at 36.0 kV and 20 mA. The chemical structures were examined from their FT-IR spectra (Bruker-TENSOR II) in the wavenumber range of 400–4000 cm^−1^. The morphologies and microstructures were investigated from SEM (JEOL JEM-220) and TEM (JEOL-2010) images, where the microscopes were operated at 15.0 and 200 kV, respectively. The NMR measurements were carried out on a 600 MHz SS-NMR JEOL ECZ600R/S3 spectrometer equipped with a 14.09 T superconducting magnet and a 3.2 mm double-resonance magic angle spinning (MAS) probe (JEOL Resonance Inc., Japan).^29^ Si-NMR spectra were obtained at 119.20 MHz. The MAS spinning speed for ^29^Si MAS was set to 12 kHz. On the basis of the following equation,^21^ as follows in eqn (1):1
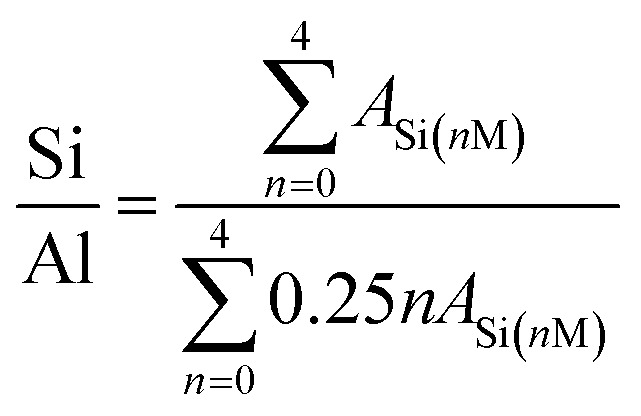
where, M represents Al, and *A* is the peak area of the fitted NMR spectrum, and *n* is Si(*n*Al), the molar ratio of the framework Si/Al was calculated.

The TG-DSC profiles were recorded on a PerkinElmer Pyris I thermal analyzer using 10 mg samples. The tests were conducted in an air atmosphere with a gas flow rate of 20 mL min^−1^ from room temperature to 900 °C at a heating rate of 5 °C min^−1^. The N_2_ adsorption–desorption isotherm at −196 °C (liquid nitrogen) was recorded using a JWGB jw-bk300 specific surface and aperture analyzer. Prior to measurement, all samples were degassed under high vacuum at 120 °C for 6 h. The Brunauer–Emmett–Teller (BET) method was used to analyze the surface area, while the Barrett–Joyner–Halenda (BJH) model was employed to calculate the mesopore size distribution and the Horvath–Kawazoe (HK) model was used to determine the micropore size. The static water contact angles (WCA) were measured using a Dataphysics-TP50 contact angle system (Dataphysics Co. Ltd., Germany) at room temperature with a total drop size of 2 μL. The temperature-programmed desorption of ammonia (TPD-NH_3_) was performed using a chemisorption analyzer PGA-1200 (Beijing Builder Electronic Technology Co., Ltd) equipped with a thermal conductivity (TC) detector. The pretreatment was conducted under a He atmosphere with a gas flow rate of 30 mL min^−1^ from room temperature to 500 °C for 1 h at a heating rate of 20 °C min^−1^ on 120 mg samples. The NH_3_ adsorption was carried out over 1 h at room temperature under a flow of NH_3_ (30 mL min^−1^). Subsequently, the physically adsorbed NH_3_ was removed under a flow of dried He at 100 °C for 30 min. Typical TPD experiments were carried out in the temperature range of 30–700 °C under a flow of dried He (30 mL min^−1^). The rate of heating was 10 °C min^−1^. Elemental analysis of C was carried out using an Elemental Vario MACRO cube. UV-Vis absorbance spectra were measured using a Shimadzu UV-2600 spectrophotometer.

### Photocatalytic degradation experiments

The photocatalytic degradation of CV dye in aqueous solution was carried out at room temperature. The light source used was a high pressure UV-Hg source (60 W) enclosed in a rectangular steel box, while the calibration of the UV-irradiation source was carried out *via* iodide–iodate actinometry and the UV-fluence rate was about 140.2 mW cm^−2^ 100 mL of a CV dye solution with a concentration of 15 ppm was taken up in a 250 mL glass beaker and 0.05 g of photocatalyst was added. Aliquots of the used sample were taken at regular intervals, centrifuged to remove any suspended solid particles at 2000 rpm and then analyzed using a UV-Vis spectrophotometer. Each experiment was carried out in duplicate in order to validate the results.

The degradation percentages, *X* (%) of the CV dye were calculated using the following eqn (2):2
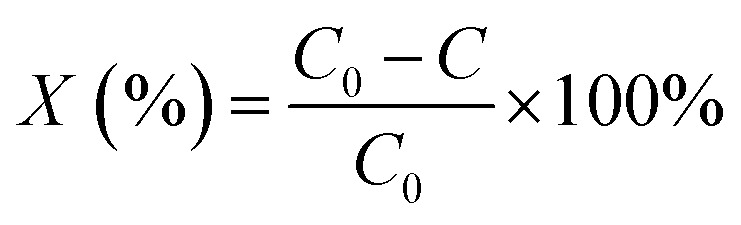
where *C*_0_ is the initial concentration of the dye and *C* is the concentration at a specified time interval, in which the standard line with a high coefficient (*R*^2^ = 0.9992) was as follows:

3 *y* = 0.1707*x* − 0.0094

## Results and discussion

### Structure characterization of hydrophobic CP

**Fig. 1:**
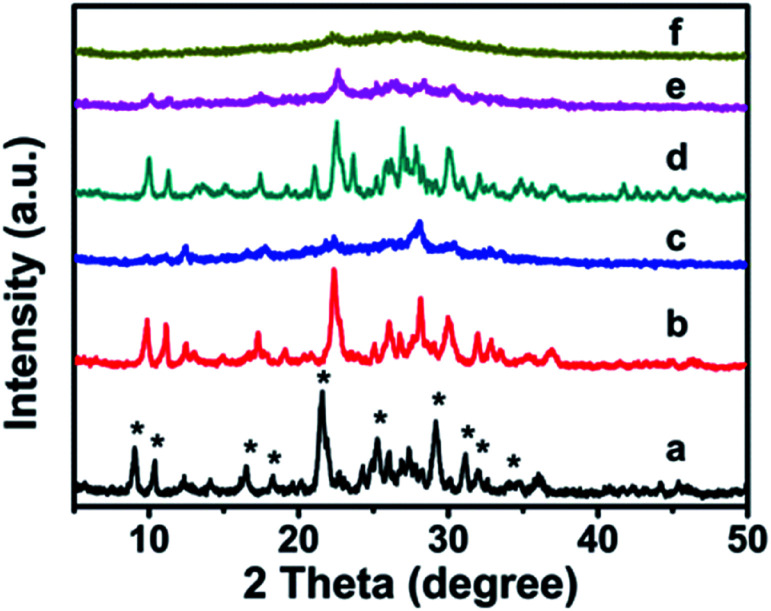
XRD patterns of the modified CP upon adding silane coupling agents. (a) CP-150-5, (b) CP-150-0.4M5, (c) CP-150-0.8M5, (d) CP-150-3, (e) CP-150-0.4D3, and (f) CP-150-0.8D3.

The XRD patterns of synthetic CP with additive amounts of the silane coupling agents MTS or DMTS are shown in Fig. 1. As can be seen, both CP-150-*x*D3 and CP-150-*x*M5 present the characteristic diffractive peaks of the HEU structure,^22^ such as [020], [200], [111], [13-1], [330], [22-2], [42-2], [350], [62-1], and [061], suggesting the presence of the typical essential frameworks after modification.

However, as seen in Fig. 1a, comparing pattern a of pure CP (CP-150-5) with pattern c of CP-150-0.8M5 indicates that the diffraction intensity of the characteristic peaks in the patterns, such as 2*θ* = 9.8° and 22.4°, obviously declined upon increasing the MTS amount from 0–0.8 mL. A similar phenomenon was also observed for the additive DMTS (as shown in Fig. 1 from d to f). These observations could be interpreted by the fact that the hydrophobic –CH_3_ in MTS or DMTS used as terminal groups play roles in slowing the crystallization procedure in the one-step synthesis system. Serrano *et al.*^23,24^ also found that the silane coupling agent slowed the crystallization of ZSM-5 *via* silanization of the protozeolitic units. Hence, the results suggest that the introduction of hydrophobic –CH_3_ groups to CP has little effect on the phase structure of HEU structures, but decreases its crystallinity.

**Fig. 2:**
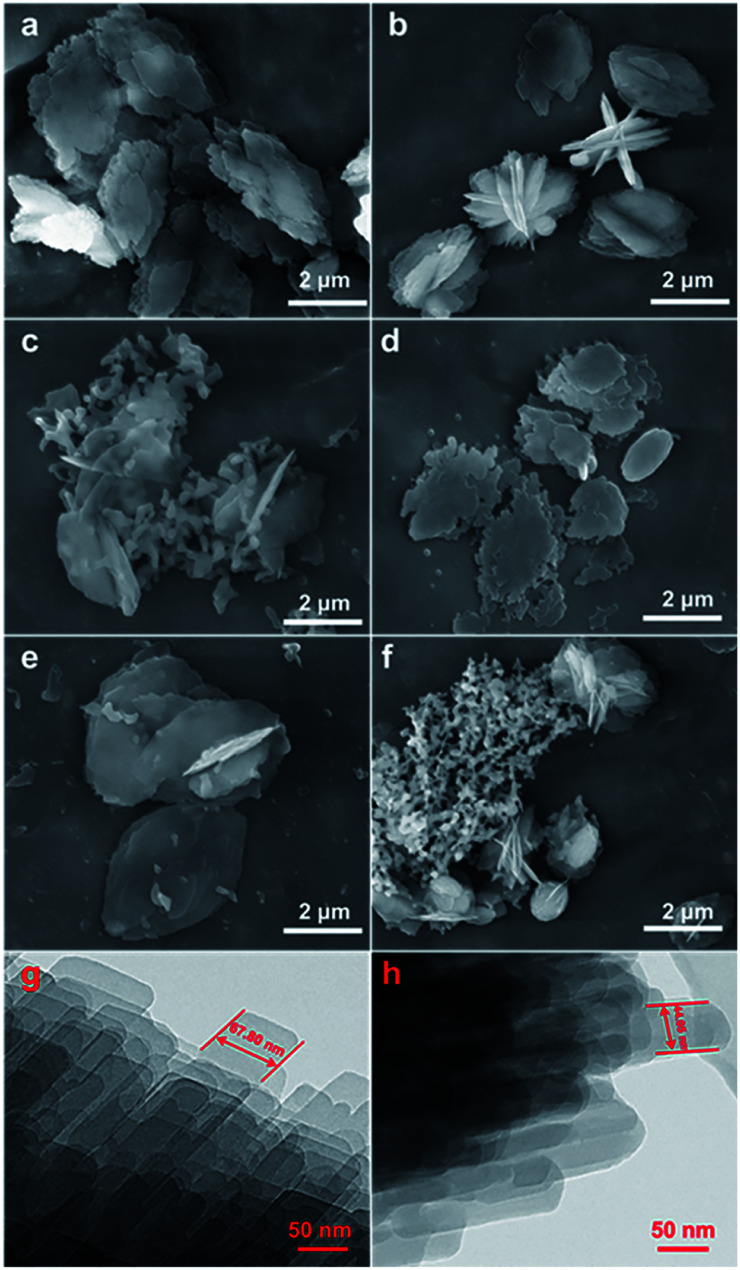
SEM images of CP modified by the addition of silane coupling agents. (a) CP-150-5, (b) CP-150-0.4M5, (c) CP-150-0.8M5, (d) CP-150-3, (e) CP-150-0.4D3, and (f) CP-150-0.8D3. The corresponding TEM images: (g) CP-150-5 and (h) CP-150-0.4M5.

**Fig. 3:**
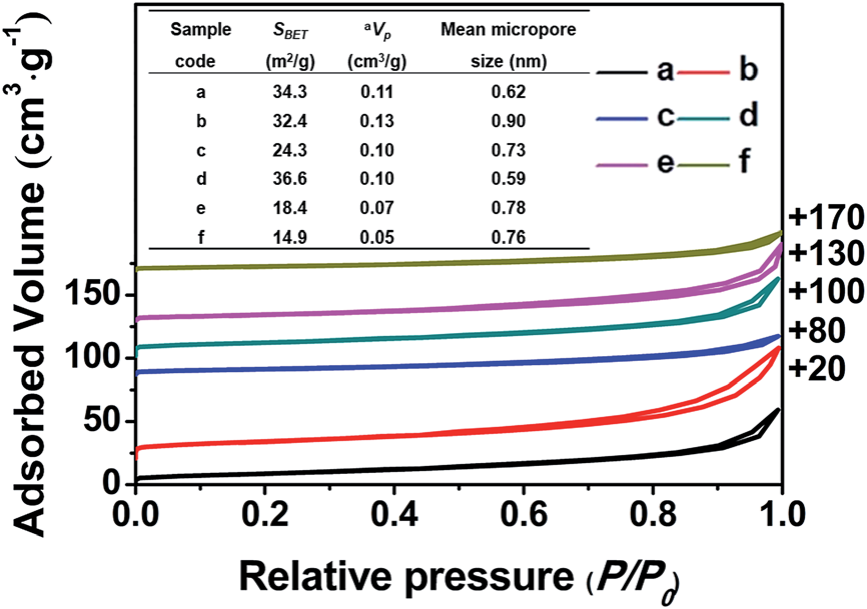
N_2_ adsorption-desorption isotherms of the samples and a summary of their textural parameters (inset Table). (a) CP-150-5, (b) CP-150-0.4M5, (c) CP-150-0.8M5, (d) CP-150-3, (e) CP-150-0.4D3, and (f) CP-150-0.8D3. *a*: estimated from the amounts adsorbed at a relative pressure (*P*/*P*_0_) of 0.9.

Fig. 2 shows the evolution of the morphology of the modified CP samples from their SEM and TEM images, taking CP-150-*x*D3 and CP-150-*x*M5 as examples, which exhibited that all of the samples had traditional layered structures. First, Fig. 3a and d present that both CP-150-5 and CP-150-3 have neat lamella morphologies in the size range of 4–5 μm, which were synthesized in the absence of a silane coupling agent. Comparably, with an increase in the amount of MTS additive, the lamellar particles of hydrophobic CP presented a decreased size of 3 μm for CP-150-0.4M5, with pellet-shaped fragments (Fig. 2b), and 1.5 μm for CP-150-0.8M5, with a large number of additional spherical and even fiber-like morphologies (Fig. 2c). These phenomena can also be observed by adding an amount of DMTS, where the lamellar size was decreased to 3 μm for CP-150-0.4D5 (Fig. 2e) and 1.5 μm for CP-150-0.8D5 (Fig. 2f). The decreases in lamellar size for hydrophobic CP (as shown in Fig. 2b and e) may be due to the thinness of the lamellar structures,^25^ which was confirmed from further TEM images. Taking CP-150-5 as an example, the lamellar size decreased from 67.8 nm (Fig. 2g) to 44.1 nm for CP-150-0.4M5 (Fig. 2h). However, the presence of a greater number of spherical particles, as shown in Fig. 2c and f, verified the appearance of amorphous aluminosilicates. Gies *et al.*^26^ also observed similar results after investigating the interlayer expansion of layered microporous silicates.

Fig. 3 shows the nitrogen adsorption–desorption isotherms of all of the related samples and their textural parameters (inset), in which the data towards the right of the plots indicate where the adsorption–desorption isotherms shift upward. As can be seen, the isotherms of all of the samples exhibit characteristic type-IV curves with a H1-type hysteresis loop at 0.8 < *P*/*P*_0_ < 0.98 due to the presence of nanoporous structures, which originated from the accumulation of the intra-spherical particles, similar to mesoporous SiO_2_ reported in the literature.^27–29^ The adsorption branches of all of the samples are basically the same, first showing increased and then decreased profiles upon the addition of the silane coupling agents.

Meanwhile, Fig. 3 (inset) shows that the BET surface areas of the hydrophobic CPs (CP-150-0.8M5 and CP-150-0.8D3) are much lower than those of the pure CPs (CP-150-5 and CP-150-3), which is due to the presence of a greater amount of amorphous aluminosilicates (as shown in Fig. 2c and f). The mean micropore sizes were obtained on the basis of the HK model (not shown), and presented obvious increasing trends from 0.62 nm for CP-150-5 or 0.59 nm for CP-150-3 to 0.73 nm for CP-150-0.8M5 or 0.76 for CP-150-0.8D3, because of the increased layer spacing arising as a result of the silanization by MTS or DMTS.^26,30^ These phenomena infer the successful modification of the CPs, similar to that reported by Sanaeepur *et al.*^31^ However, the mean mesopore sizes were around 3.1–3.4 nm (corresponding to a *P*/*P*_0_ value of approximately 0.42 in the nitrogen adsorption–desorption isotherms) on the basis of the BJH model (not shown), which may be associated with tensile strength effects.^32–35^

**Fig. 4:**
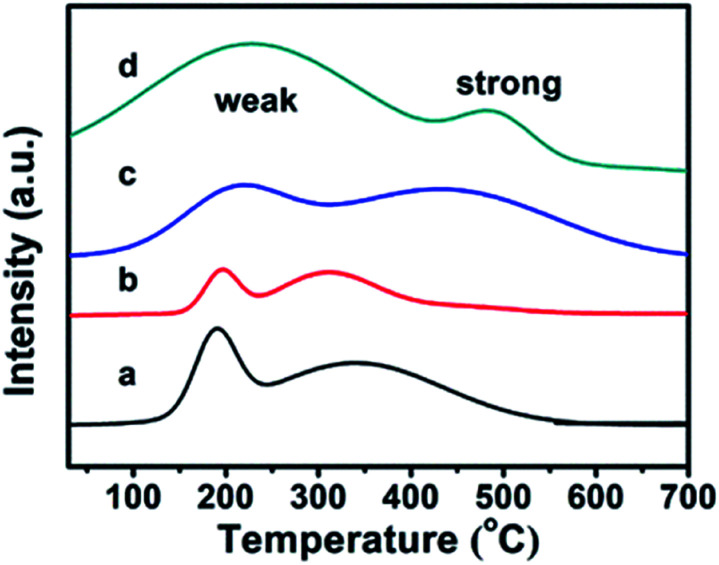
TPD-NH_3_ profiles for (a) CP-150-5, (b) CP-150-3, (c) CP-150-0.4M5, and (d) CP-150-0.6D3.

The CPs before and after modification were investigated using the TPD-NH_3_ method, and their thermodesorption curves of ammonia are shown in Fig. 4. Although the appearances of Brønsted and Lewis acid sites in all samples were not obviously distinguished,^36^ the qualitative evaluation of the acid site strength could be justified: the low temperature peak in the region of 100–350 °C is due to ammonia desorption from weak acid sites, while the high temperature peak in the 350–700 °C region corresponds to ammonia desorption from strong acid sites. In detail, the pure CPs (CP-150-3 and CP-150-5) reveal almost the same strength of weak acid (190 °C) and strong acid (330 °C) sites in the TPD-NH_3_ profiles, corresponding to acid amounts of around 0.1156 and 0.2537 mmol NH_3_ per g, respectively. As compared in Fig. 4a and b, the peak position of the weak and strong acid sites moved to a higher temperature (210 °C and 430 °C) for CP-150-0.4M5 (Fig. 4c), also, the acid amounts were increased to 0.3109 and 0.6662 mmol NH_3_ per g. In particular, CP-150-0.6D3 (Fig. 4d) shows stronger acid sites (230 °C and 490 °C) and larger acid amounts (1.3171 and 0.3054 mmol NH_3_ per g). These results suggest that the modifications of the CPs using a silane coupling agent may be beneficial for enhancing the strengthen and amounts of the acid sites, which could be useful for acid–base catalysis.

### Influencing factors on the synthesis of the hydrophobic CPs

In order to synthesize hydrophobic CPs with high purity, the effects of the crystallization temperature and time on the HEU structures and crystal phases were investigated. As shown in Table 1, the appearances of amorphous phase occurred at less than 140 °C or within 2 days, while mordenite or phillipsite were obtained at higher than 150 °C or over 6 days.

**Fig. 5:**
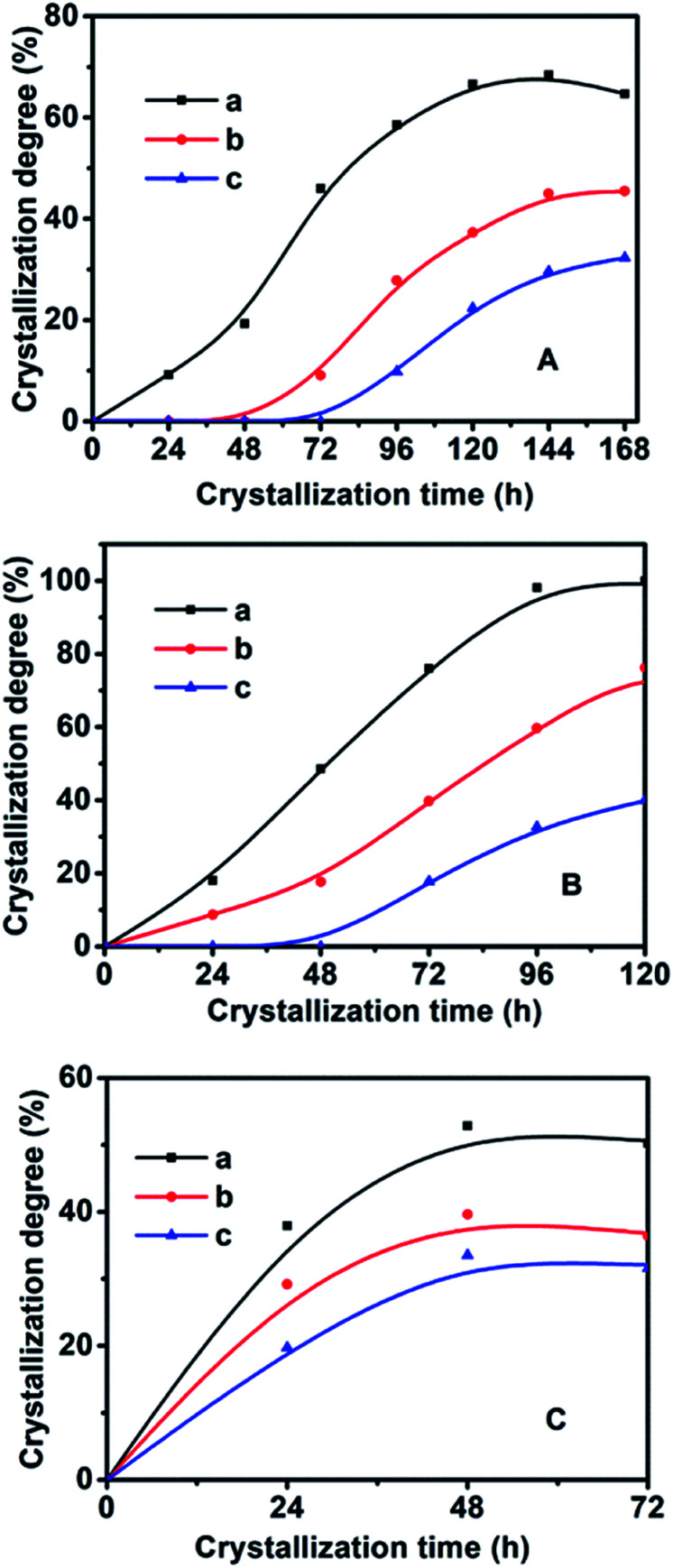
Crystallization kinetic profiles of the related CPs using MTS or DMTS as a silane coupling agent at A (140 °C), B (150 °C), and C (180 °C). (a) Pure CP without the addition of any MTS or DMTS, (b) CP-0.4M, and (c) CP-0.4D.

On the basis of the XRD patterns of the CP samples synthesized using MTS or DMTS as a silane coupling agent at different crystallization temperatures for different crystallization times, the degree of crystallinity of the synthesized CPs was calculated from the relative values of the sum of the intensities of ten peaks for the HEU structures:^22^ [020], [200], [111], [13-1], [330], [22-2], [42-2], [350], [62-1] and [061], in which, the value of a fully crystallized sample (CP-150-5) was normalized to 100%. In order to investigate the influence of DMTS and MTS on CP crystallization, Fig. 5 illustrates the crystallization kinetic curves of synthetic pure CP, CP-0.4M and CP-0.4D at different crystallization temperatures. The induction time (*t*_0_) is defined as the crystallization time that elapses to achieve a crystallinity of about 15%, which involves relaxation (hydrolysis and polycondensation of aluminosilicate clusters), nucleation (nuclei formation), and transition (slow growth of nuclei to a detectable size).^6^

The activation energy (*E*_*n*_) and frequency factor (*A*_*n*_) values for the induction stage are dependent on the crystallization temperature according to the Arrhenius equation, meanwhile, the apparent activation energy (*E*_g_) values of the growth period and their rate constants (*k*_max_) could also be calculated.

As seen in Fig. 5 and in Table 2, the *t*_0_ value at crystallization temperature of 150 °C was maintained at this value from 22 h for the synthetically pure CP to 44 h for a MTS-modified CP (CP-0.4M), to 69 h for a DMTS-modified CP (CP-0.4D), the reason being that the presence of hydrophobic methyl groups inhibits the growth of the nucleus.^20,21^ Meanwhile, as shown in Table 2, significant *t*_0_ values for the induction time were observed at increased crystallization temperatures. Taking CP-0.4M as an example, from 78 h at 140 °C to 44 h at 150 °C, and even 14 h at 180 °C, which may be due to the fact that the solubility of the Si and Al sources in the induction stage is generally enhanced by increasing the temperature, in good agreement with the results of Joshi *et al.*,^37^ as follows:

**Table 2:** A summary of the *E*_*n*_, *t*_0_, and *k*_max_ values during CP crystallization

Sample	*T* (°C)	*E*_*n*_	ln *A*_*n*_	*t*_0_ (h)	Growth periods		
					*k*_max_	*E*_g_	ln *A*_g_
Pure CP	140	48.1	10.5	36	1.11	13.2	4.0
	150			22	1.27		
	180			10	1.58		
CP-0.4M	140	65.5	14.8	78	0.78	16.5	4.6
	150			44	0.94		
	180			14	1.22		
CP-0.4D	140	69.5	15.5	106	0.52	17.0	4.3
	150			69	0.62		
	180			18	0.82		

The values of *E*_*n*_ (kJ mol^−1^) and *A*_*n*_ for the induction stage are determined from the nucleation rate (1/*t*_0_) dependence on the temperature, based on the following Arrhenius equation:4
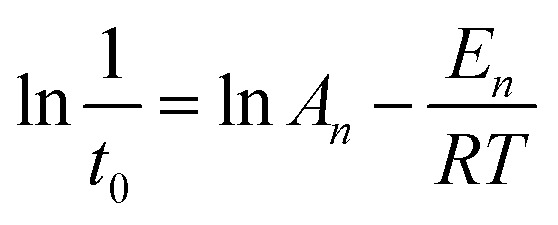


The Arrhenius equation is used to calculate the *E*_g_ (kJ mol^−1^) of the growth period, as shown in the equation. The rate constant (*k*_max_) can be obtained from the slope of the line between the two points with the biggest increase:5
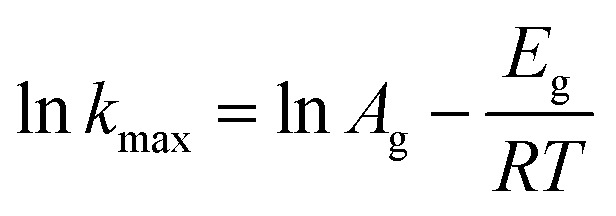
where *R* is the ideal gas constant, *T* is the absolute temperature (K), and *A*_g_ is the frequency factor for the growth stage.

The nucleation rate is usually considered as the reciprocal of the induction time. In addition, as can be seen in Table 2 and Fig. S2 of the ESI† section, the *E*_*n*_ value of pure-CP (48.1 kJ mol^−1^) is much lower than those of CP-0.4M (65.5 kJ mol^−1^) and CP-0.4D (69.5 kJ mol^−1^). After the induction periods, the rapid growth led to an abrupt change in the slope of crystallization behaviour, leading to increased *k*_max_ values with improved crystallization temperature, taking CP-0.4M as an example, from 0.78 at 140 °C to 0.94 at 150 °C, and even 1.22 at 180 °C. However, as shown in Table 2, after modification upon the addition of MTS and DMTS, the *k*_max_ values decreased from 1.58 for the synthetically pure CP to 1.22 for CP-0.4M, and 0.82 for CP-0.4D. In particular, as can be seen in Table 2 and Fig. S3 of the ESI† section, the *E*_g_ value of the synthetically pure CP (13.2 kJ mol^−1^) is lower than those of CP-0.4M (16.5 kJ mol^−1^) and CP-0.4D (17.0 kJ mol^−1^). Additionally, the overall *E*_g_ values of all samples were much lower than the *E*_*n*_ values, suggesting that the induction period is kinetically controlled, while the rapid growth process is thermodynamically controlled.

### The enhanced hydrophobicity

Fig. S4† shows the surface hydrophobicity profiles of the modified CPs on the basis of WCA measurements. As can be seen, the synthetically pure CP (CP-150-5) exhibits a WCA value of *ca.* 14.1° (Fig. S4-a†), comparably, the hydrophobic-modified CPs reveal much larger WCA values of 40.2° for CP-150-0.4M5 (Fig. S4-b†) and 54.7° for CP-150-0.6M5 (Fig. S4-c†) when using MTS as the silane coupling agent. Similarly, when using DMTS as the silane coupling agent, the values were from 13.2° for CP-150-3 (pure CP) (Fig. S4-d†) to 55.2° for CP-150-0.4D3 (Fig. S4-e†) and 70.8° for CP-150-0.6D3 (Fig. S4-f†). These phenomena clearly indicate the strengthened hydrophobicity that arises on increasing the amount of coupling agent,^38^ further proving the successful hydrophobicity of the synthetic CP.

**Fig. 6:**
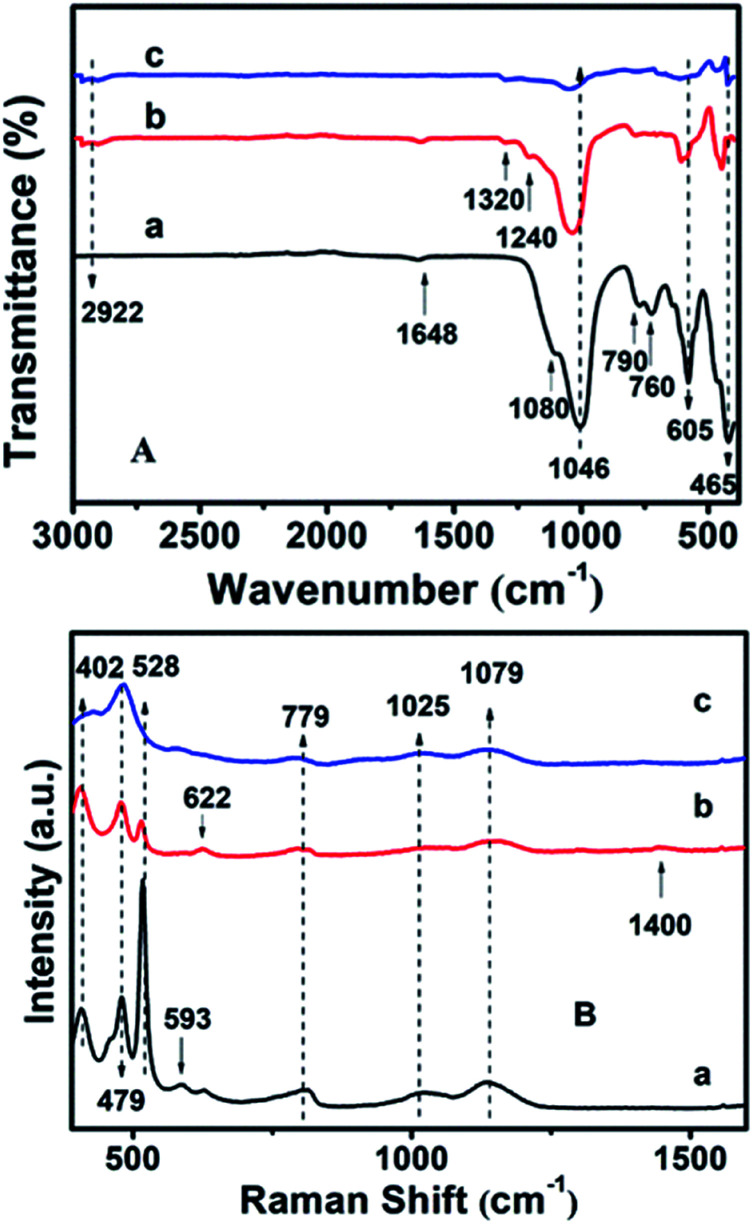
(A) FT-IR and (B) UV-Raman spectra of (a) CP-150-5, (b) CP-150-0.4M5, and (c) CP-150-0.8M5.

The chemical groups in the hydrophobic CP could be further verified by FT-IR measurements. In Fig. 6A-a, the absorption peaks at about 443, 760, 790 and 1080 cm^−1^ correspond to Si–O–Si bands, while the band at 1046 cm^−1^ is due to the –OH stretching vibration in the Si–OH groups, whereas another at 1648 cm^−1^ can be attributed to the vibration of –OH groups derived from adsorbed water.^39–41^ Meanwhile, the absorption peak at about 465 cm^−1^ can be assigned to the bending vibration of T–O (T = tetrahedral site) inside the tetrahedron,^42^ while another near 605 cm^−1^ is related to the outer tetrahedral double loop.^43^ Comparably, Fig. 6A-b clearly shows that the additional absorption peak in the vicinity of 1240 cm^−1^ can be assigned to a Si–CH_3_ band, and the extra peaks at 2922 and 1320 cm^−1^ are ascribed to the C–H vibration of the methyl group, implying the strengthened hydrophobicity after MTS modification, in good agreement with the WCA analysis of the behavior of the amount of MTS added (Fig. S4†). However, the absorption peaks are in good agreement with the results of the XRD patterns (Fig. 2c) and SEM images (Fig. 3c).

Furthermore, the additional peak at 793 cm^−1^ in Fig. 6A-c usually represents the stretching vibration of the O–T–O group,^42^ suggesting the presence of a quartz impurity in CP-150-0.8M5. The hydrophobic CPs using DMTS as a silane coupling agent also presented similar FT-IR spectra.

The UV-Raman spectra in the T–O–T bending region are very sensitive to the ring systems in the presence of the CP framework, which are shown in Fig. 6B for hydrophobic CP using MTS as a silane coupling agent. As can be seen in Fig. 6B-a, pure CP (CP-150-5) exhibits main peaks at 479, 528, 593, 779, 1025 and 1079 cm^−1^, respectively. In detail, the bands centered at near 479 and 779 cm^−1^ are the characteristic peaks of monomeric silicate,^43^ while the others at 593 and 1025 cm^−1^ are the characteristic peaks of oligosilicate.^43^ Meanwhile, the peak at 1079 cm^−1^ can be attributed to the stretching vibration of the Si–O bond at the end of the double quaternary ring,^44^ while the peak at 528 cm^−1^ and the Raman band at 402 cm^−1^ correspond to T–O as the anti-symmetric stretching vibration and distorted six-membered rings, respectively.^44^ In addition, the appearance of a weak characteristic peak at around 620 cm^−1^ suggested that a small amount of aluminate did not bind to silicate. Fig. 6B-b provides the microstructure information of MTS-modified CP (CP-150-0.4M5), in which the peak intensity at 479 and 779 cm^−1^ are weaker than that of CP-150-5 (Fig. 6B-a), suggesting low crystallinity, in good agreement with the analysis of the XRD patterns (Fig. 1b). The unobvious peaks at 1079, 1025, and 593 cm^−1^ seem to be related to fewer double quaternary rings, indicating no oligomeric silica roots in CP-150-0.4M5. However, an extra peak at 1400 cm^−1^ can be attributed to –CH_2_ scissoring or –CH_3_ deformation,^45^ again indicting the successful hydrophobicity of CP by MTS modification, consistent with the results of the mentioned-above characterization and discussion. Notably, the peak at 402 cm^−1^ is very weakened in spectrum of CP-150-0.4M5 (Fig. 6B-b), and even disappeared in that of CP-150-0.8M5 (Fig. 6B-c), suggesting the strong impact that methyl groups have on the distortion of the six-membered rings. In addition, we noticed that the monomer silicate radical at 779 cm^−1^ was shifted towards a low wavenumber upon the addition of an increased amount of MTS, which may be due to the expansion of the lattice caused by the introduction of methyl groups.

**Fig. 7:**
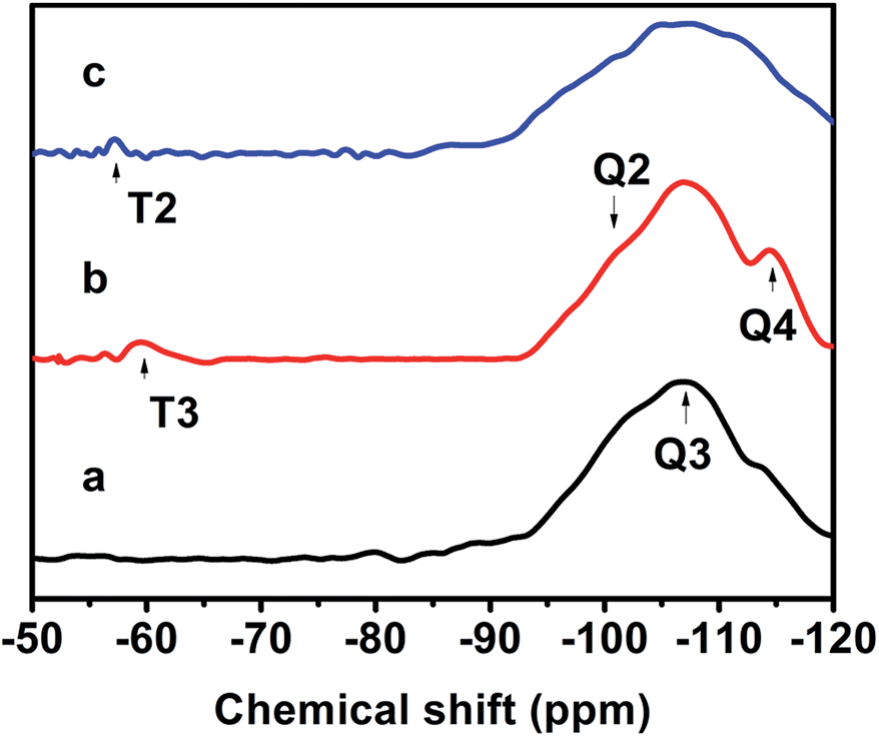
^29^Si-NMR spectra of (a) CP-150-5, (b) CP-150-0.4M5, and (c) CP-150-0.6D3.

Fig. 7 exhibits the ^29^Si MAS NMR spectra, which further provide detailed information about the coordination of the Si atom environments in the hydrophobic CPs. As can be seen, all of the samples present strong resonance signals at −113/−109, −104, −99, −95 ppm, and a weak signal at −87 ppm, corresponding to Si(0Al) (Q_4_), Si(1Al) (Q_3_), Si(2Al) (Q_2_), Si(3Al) (Q_1_) and Si(4Al) (Q_0_) sites,^46^ respectively. On the basis of the mentioned-above eqn (1), the molar ratio of the framework Si/Al was calculated, as follows: 5.02, 5.55, and 5.45, corresponding to CP-150-5, CP-150-0.4M5, and CP-150-0.6D3, respectively. These results confirm that silanization could be useful for improving the Si/Al ratio, similar to the results determined by Tanaka *et al.*^47^ Further evidence for the successful silylation of the obtained CP can also be seen in Fig. 7b and c. In which, the additional two signals at −57, and −62 ppm were observed, indexed as T2, and T3, corresponding to units of Si(OSi)_2_R, and Si(OSi)_3_R′ units,^48^ respectively, where R is –(CH_3_)_2_ and R′ is –CH_3_. These results suggest that the methyl and dimethyl groups were successfully incorporated onto the HEU frameworks *via* a one-step method.

Fig. S5† shows the before and after TG-DSC curves of the modified CPs. As can be seen, all of the samples present two weight loss stages, including rapid dehydration at 30–300 °C and slow decomposition at 300–900 °C.^49^ In the first stage, the weight loss of CP-150-3 was around 13.1 wt% (Fig. S5-a†), significantly greater than those of CP-150-0.4M5 (4.87 wt%) (Fig. S5-b†) and CP-150-0.6D3 (3.85 wt%) (Fig. S5-c†), suggesting the effective hydrophobic performance of CP modified by a silane coupling agent. Subsequently, the weight loss of all samples levelled off in the range of 300–900 °C, showing 1.4 wt% for CP-150-3 (Fig. S5-a†), slightly greater than the values of CP-150-0.4M5 (0.7 wt%, as shown in Fig. S5-b†) and CP-150-0.6D3 (1.0 wt%, as shown in Fig. S5-c†).

Overall, the fact that the amount of water adsorbed by the modified CPs is much lower than that of pure CP suggests the successful hydrophobicity of the synthetic CPs. Also, the elemental analysis indicates that the C amount is around 0.46 wt% for CP-150-0.4M5 and 0.63 wt% for CP-150-0.6D3.

Correspondingly, the DSC profiles show an obvious endothermic peak at 120 °C for CP-150-3 (Fig. S5-a†), but a broadened and weakened endothermic peak at 30–300 °C for CP-150-0.4M3 (Fig. S5-b†), which could be attributed to the removal of physically adsorbed water. However, the modified CPs (CP-150-0.4M3 and CP-150-0.6D3) presented a slightly exothermic peak at more than 660 °C (Fig. S5-b and -c†), which can be attributed to the decomposition of the hydrophobic methyl groups. Comparably, pure CP (CP-150-3) did not reveal an obvious exothermic peak at 300–900 °C (Fig. S5-a†), showing its good thermal stability, in good agreement with our previous report.^50^

### Photocatalytic performance of the hydrophobic CPs

The XRD patterns of the ZnO/CPs as photocatalysts are shown in Fig. S6.† As can be seen, all of the samples show typical diffractive peaks of the ZnO and CP phases, in which the 2*θ* peaks at 9.8, 11.2, 22.4, 22.7, 26.1, 28.2, and 32° were assigned to the HEU structure,^19^ similar to those of the samples shown in Fig. 1. These results suggest that the HEU structures of the CPs after ZnO loading remain intact. Meanwhile, as can be seen in Fig. S6,† the presence of new peaks at 2*θ* angles of 32, 36, 38, and 58° correspond to the crystal planes [100], [002], [101] and [110], which can be indexed to a wurtzite hexagonal phase of ZnO.^51^ According to the Scherrer equation,^52^ the crystallite sizes of the loaded ZnO were estimated, giving values of 20.3 nm for ZnO/natural CP (Fig. S6-a†), 10.3 nm for 0.8-ZnO/natural CP (Fig. S6-b†), 23.4 nm for ZnO/CP-150-3 (Fig. S6-c†), 23.0 nm for ZnO/CP-150-0.4M5 (Fig. S6-d†), and 26.5 nm for ZnO/CP-150-0.4D3 (Fig. S6-e†), close to the data reported in the literature.^51^

The direct band gap energy of ZnO/CP-150-3 was determined using the Tauc equation,^53^ as shown in Fig. S7 of the ESI† section:

6 (*Ahν*)^2^ = *C*(*hν* − *E*_b_)

where *A* is the absorbance, *h* is Planck's constant, *ν* is the frequency and *E*_b_ is the band gap energy. The direct band gap energy of ZnO/CP-150-3 was found to be 3.35 eV, as shown in Fig. S7 in the ESI† section, whereas the band gap of ZnO reported in literature is 3.37 eV.^3^ The bandgap energies of TiO_2_,^53^ CdS,^54^ and CeO_2_ (ref. 55) reported in literature are 3.3, 2.45 and 2.81 eV, respectively.

**Fig. 8:**
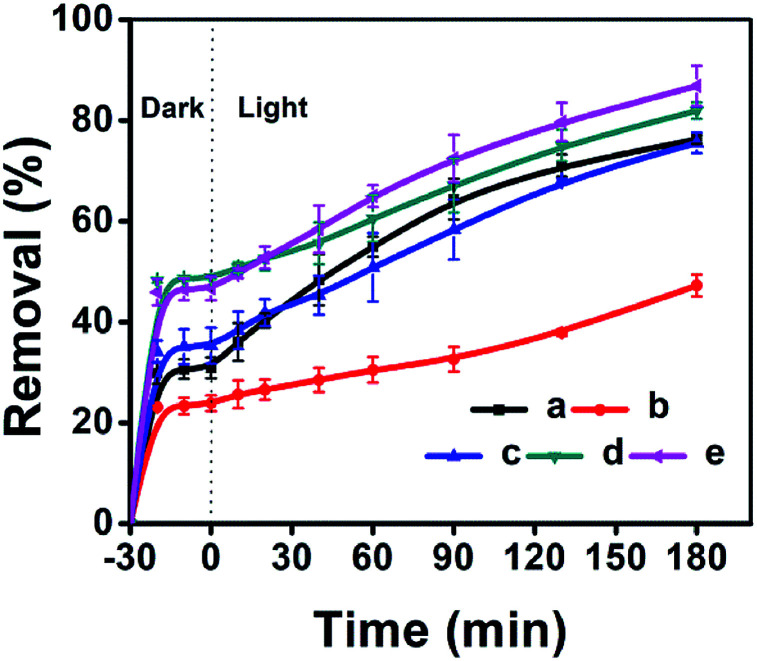
ZnO-loaded CPs as photocatalysts for the % adsorption and degradation of CV dye: (a) ZnO/Natural-CP, (b) 0.8-ZnO/natural-CP, (c) ZnO/CP-150-3, (d) ZnO/CP-150-0.4M5, and (e) ZnO/CP-150-0.4D3.

The adsorptive and photocatalytic performances for CV degradation using ZnO-loaded CPs as photocatalysts were preliminary explored. As can be seen in Fig. 8, the adsorption capacities of the used hydrophobic ZnO/CP-150-0.4M5 (Fig. 8c) and ZnO/CP-150-0.4D3 (Fig. 8d) in an aqueous solution were about 45%, more than those (30%) of ZnO/CP-150-5 (Fig. 8b) and ZnO/natural-CP (Fig. 8a) under dark conditions. Furthermore, Fig. 8b shows that the removal efficiency of CV dye (adsorption and degradation) was reduced from 78 to 47% upon increasing the loading content of ZnO. At high ZnO loading, particles may tend to aggregate, which reduces the interfacial area between the reaction solution and the photocatalyst, leading to a decrease in the number of active sites on the catalyst surface.^56^ Also, the excess of ZnO particles may mask some parts of the photosensitive surfaces and consequently hinder or even reflect light penetration. In particular, the deactivation of activated molecules in these aggregates due to collision with the ground state ZnO molecules can also reduce the extent of degradation. Hence, an optimal amount of ZnO has to be added in order to avoid unnecessary excess and also ensure the total absorption of light photons for efficient photo-mineralization.^57^

In general, the following Langmuir–Hinshelwood equation^58^ is the best model for describing the kinetic behaviour of heterogeneous photocatalytic reactions:7
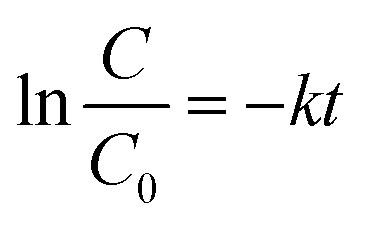
where *k* is the rate constant of photocatalysis (min^−1^), *C*_0_ is the concentration of dye solution (mg L^−1^), *C* is the concentration of dye solution at any time (mg L^−1^), and *t* is the time of photocatalysis (min).

As can be seen in Fig. S8 of the ESI† section, the *k* values of ZnO/natural-CP, ZnO/CP-150-3, ZnO/CP-150-0.4M5, and ZnO/CP-150-0.4D3 are 6.3 × 10^−3^, 5.9 × 10^−3^, 6.0 × 10^−3^ and 7.1 × 10^−3^ min^−1^, respectively, indicating that there was no significant change in the catalytic rate. Subsequently, the overall removal performances of CV dye after around 180 minutes reached up to 81.92% for ZnO/CP-150-0.4M5 and 86.82% for ZnO/CP-150-0.4D3, slightly higher than the values for ZnO/natural-CP or ZnO/CP-150-3. Obviously, it can be considered that the hydrophobic CPs have better adsorption ability for CV dye in aqueous solution than pure CP and natural CP, making them promising adsorbents for the removal of organic pollutants from aqueous solution.

## Conclusions

The addition of MTS or DMTS silane additives to modified CP samples was investigated, and thereafter hydrophobic CPs with controlled particle size were successfully prepared. Various characterization techniques demonstrated that the hydrophobicity of CP could be enhanced according to the amount of MTS or DMTS added, but, an excess of MTS (DMTS) in the synthetic systems resulted in the appearance of an impure heterophase (phillipsite), and they even became amorphous. On the basis of the Arrhenius equation, the *E*_*n*_ values obtained *via* DMTS and MTS modification were around 69.5 and 65.5 kJ mol^−1^, respectively, greater than that of pure CP (48.1 kJ mol^−1^), while their induction time (*t*_0_) at the same crystallization temperature was increased compared to the value of pure CP. In particular, the *E*_g_ value was much lower than the *E*_*n*_ value. These results indicate that the induction process is a controlled step during the hydrothermal synthetic system, in which the effect of the additive MTS (or DMTS) not only slows down the formation of crystal nuclei, but also decreases the particle sizes. The strong impact that methyl groups have on the distortion of the six-membered rings was obvious in the induction periods, leading to the expansion of the lattice of CP and an enhanced Si/Al ratio. The removal of CV dye from aqueous solution using the hydrophobic CPs as supports and ZnO as a photocatalytic species showed better performance over natural CP and pure CP supports. These results might provide useful information for the design of advanced hydrophobic supports for potential applications in wet environments.

## Conflicts of interest

There are no conflicts to declare.

## Supplementary Material

RA-010-D0RA03151H-s001
