# Supplementary material for: One-step synthesis of hydrophobic clinoptilolite modified by silanization for the degradation of crystal violet dye in aqueous solution
Source: RSC Adv. 2020 Jun 15;10(38):22809–18. doi: 10.1039/d0ra03151h (PMC9054683; doi:10.1039/d0ra03151h)
Supplement: RA-010-D0RA03151H-s001 [file RA-010-D0RA03151H-s001.pdf]

Electronic Supplementary Material (ESI) for RSC Advances.
This journal is © The Royal Society of Chemistry 2020

One-step synthesis of hydrophobic clinoptilolite modified by silanization for degradation of crystal violet dye in aqueous solution

Jian Jiao, Jihong Sun *, Raza Ullah, Shiyang Bai* and Chengwei Zhai

Beijing Key Laboratory for Green Catalysis and Separation, Department of Chemistry and Chemical Engineering, Beijing University of Technology, Beijing, 100124, China

Electronic Supporting Information

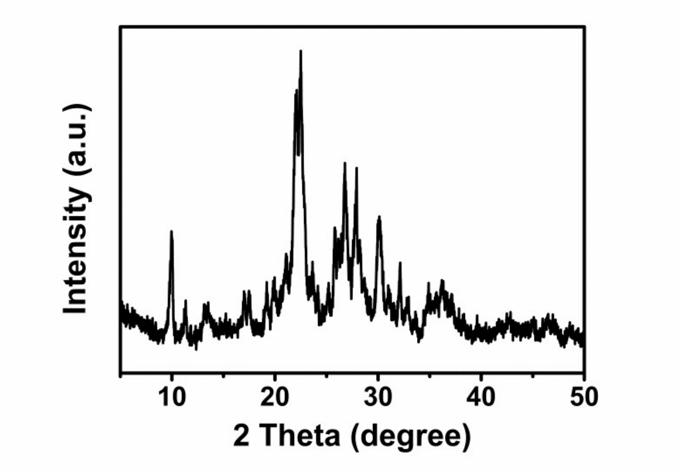

Fig. S1 XRD pattern of natural CP, used as seed.

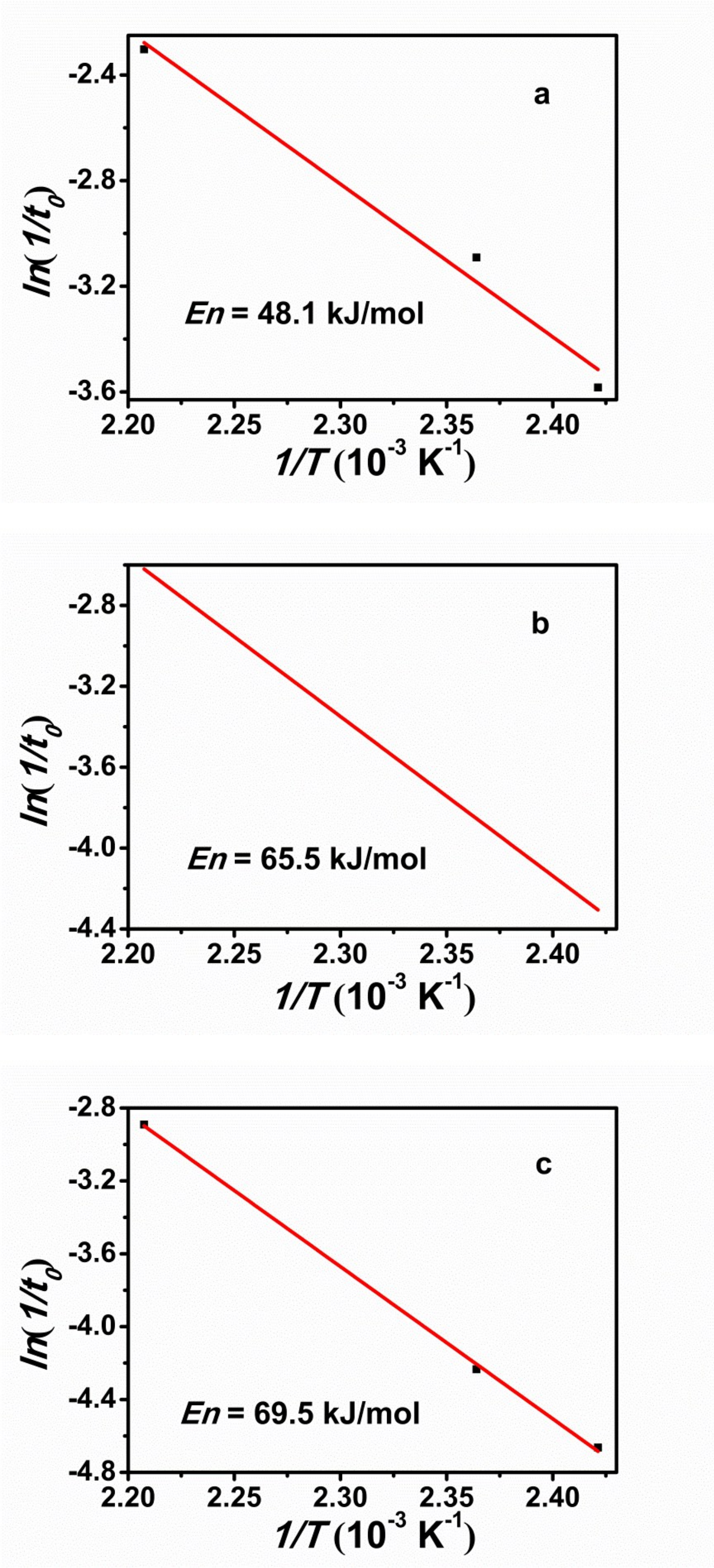

Fig. S2 Arrhenius plots of induction time for (a) synthetic pure CP, (b) CP-0.4M, and (c) CP-0.4D.

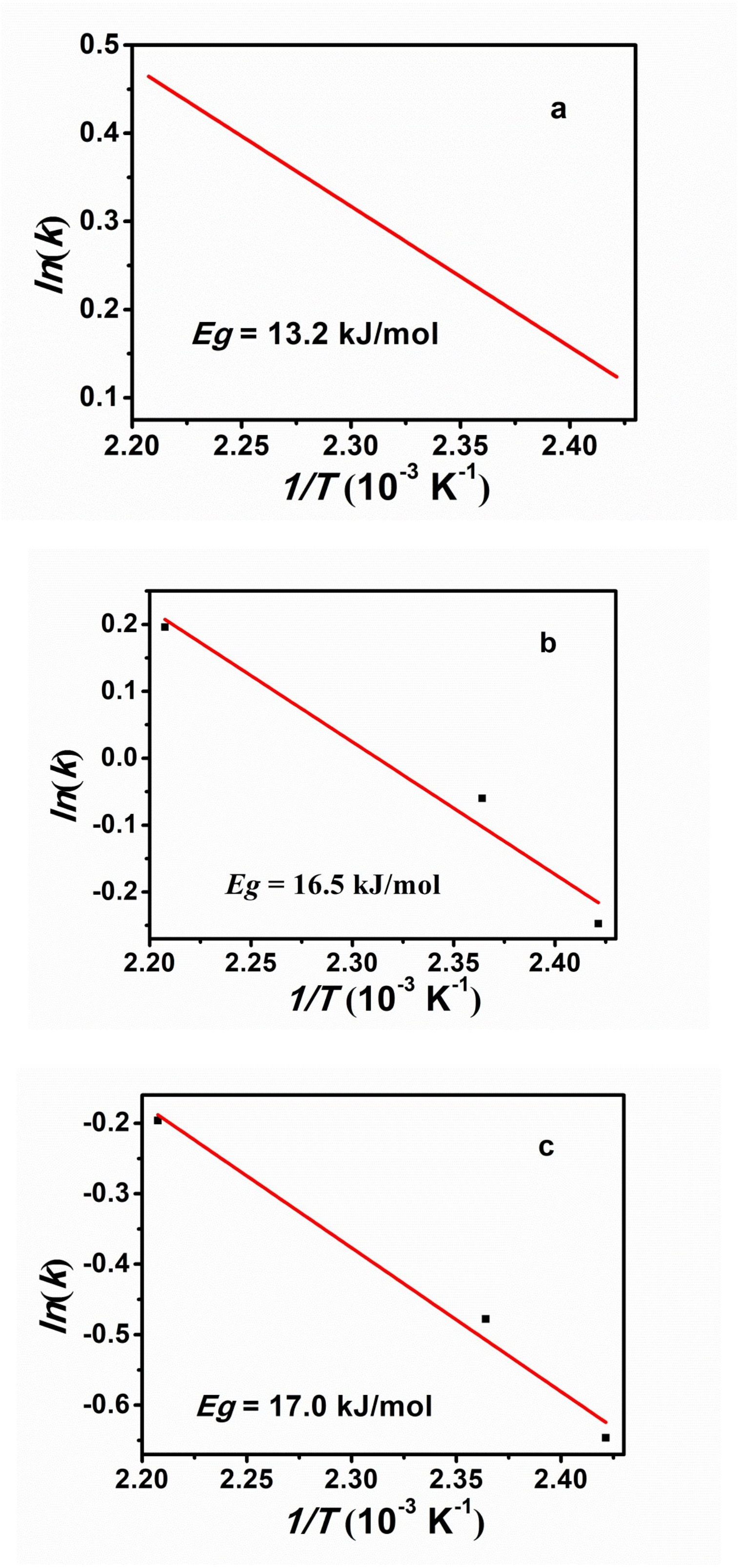

Fig. S3 Arrhenius plots of growth periods for (a) synthetic pure CP, (b) CP-0.4M, and (c) CP-0.4D.

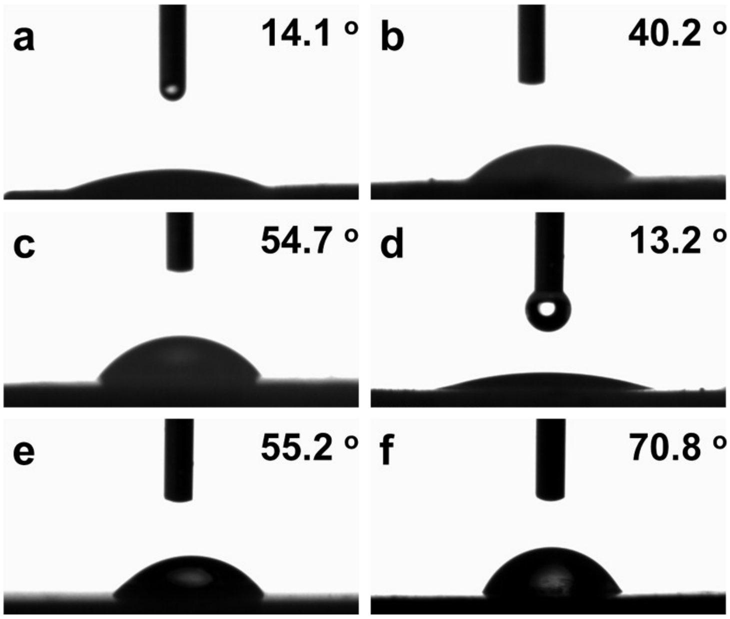

Fig. S4 WCAs illustrations of (a) CP-150-5, (b) CP-150-0.4M5, (c) CP-150-0.6M5, (d) CP-150-3, (e) CP-150-0.4D3, and (f) CP-150-0.6D3.

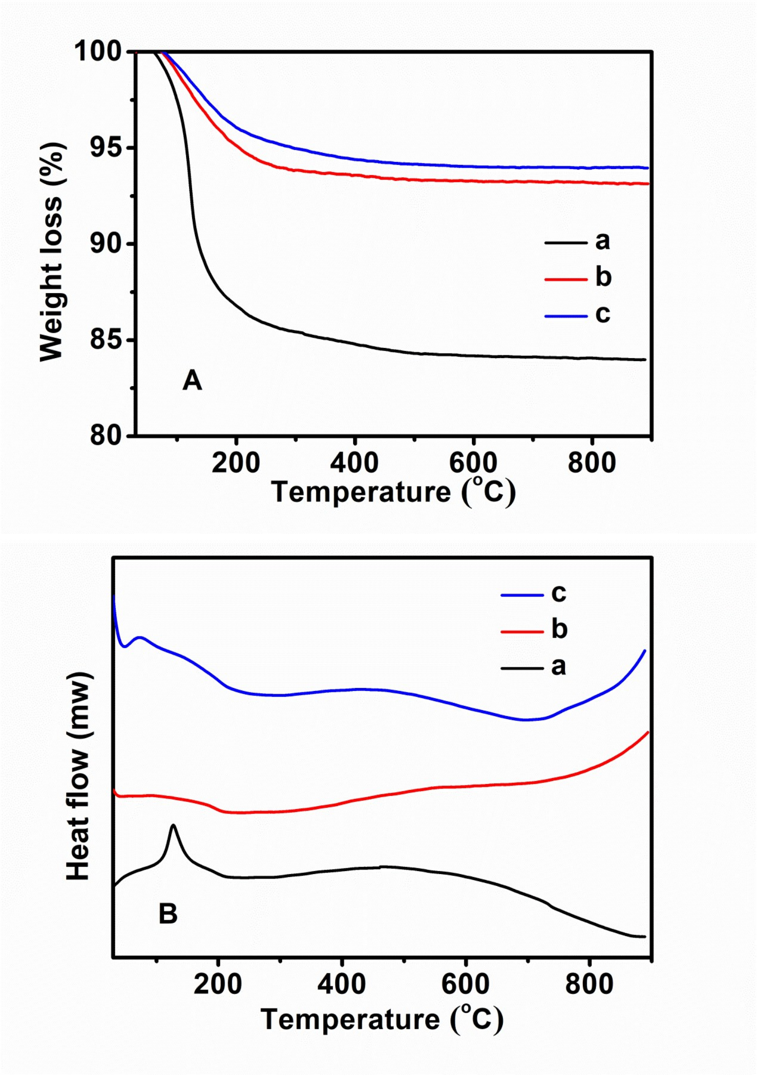

Fig. S5 TG (A)and DSC (B) profiles of as-synthesized samples: (a) CP-150-3, (b) CP-150-0.4M5, and (c) CP-150-0.6D3.

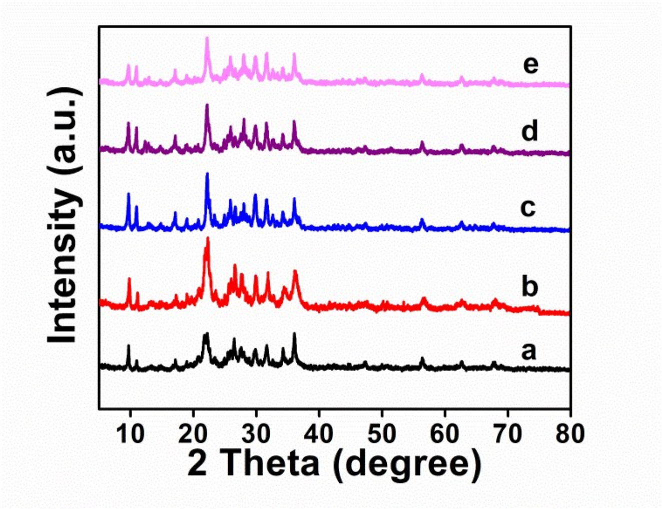

Fig. S6 XRD patterns of (a) ZnO/natural CP, (b) 0.8-ZnO/natural CP, (c) ZnO/CP-150-3, (d) ZnO/CP-150-0.4M5 and (e) ZnO/CP-150-0.4D3.

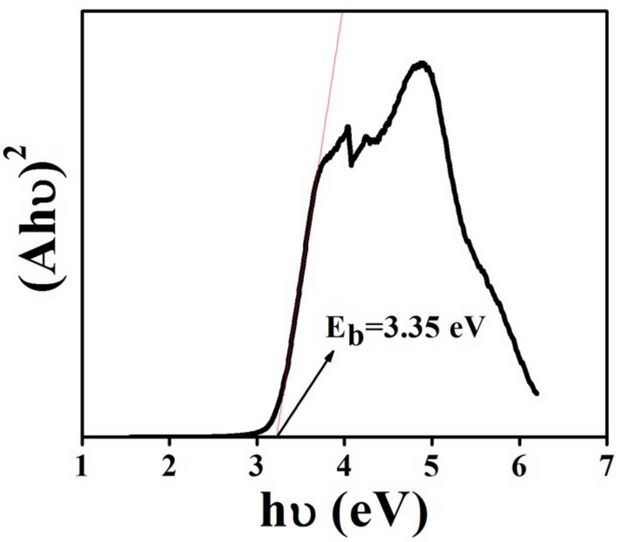

Fig. S7 Band gap energy of ZnO/CP-150-3 determined using Tauc relation.

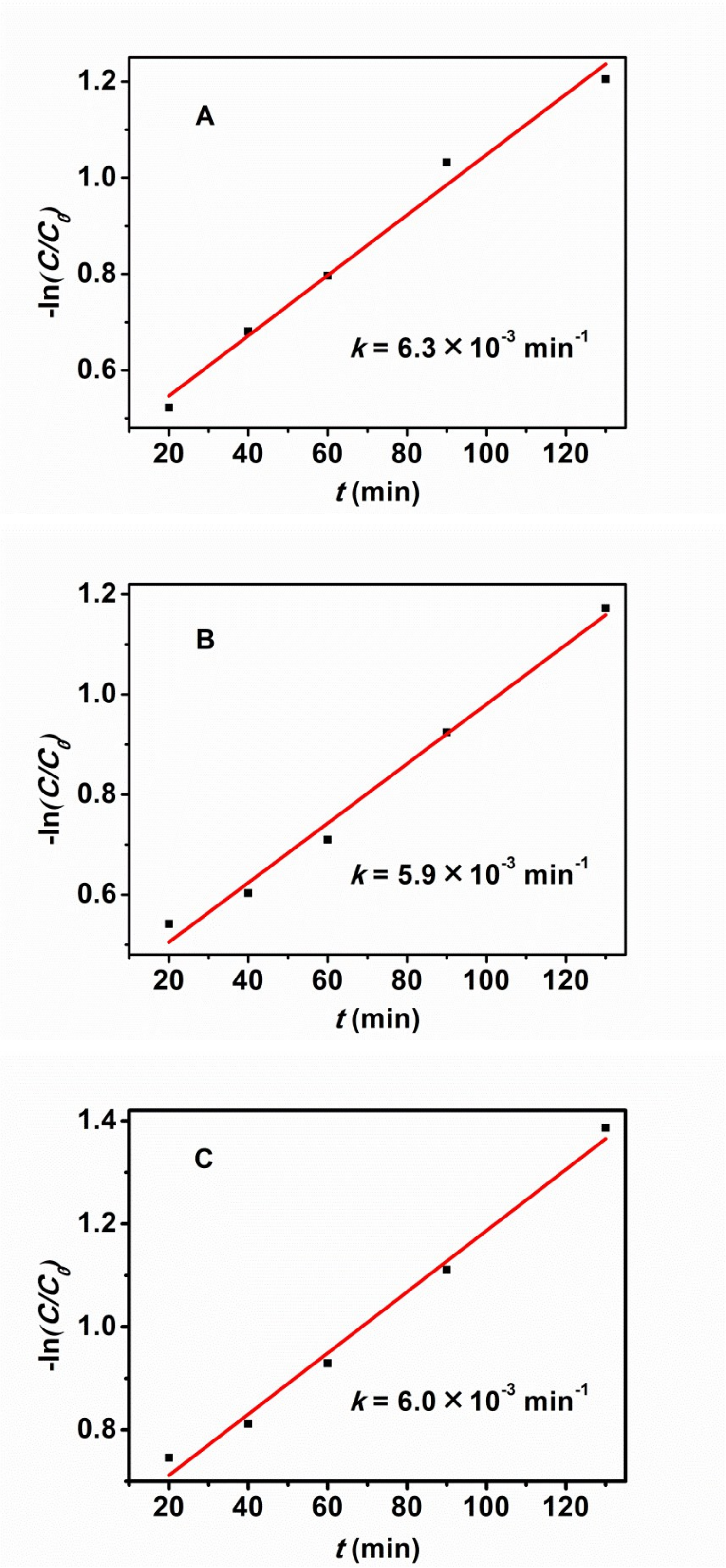
A
k = 6.3 × 10-3 min-1
B
k = 5.9 × 10-3 min-1
C
k = 6.0 × 10-3 min-1
-ln(C/C0)
t (min)

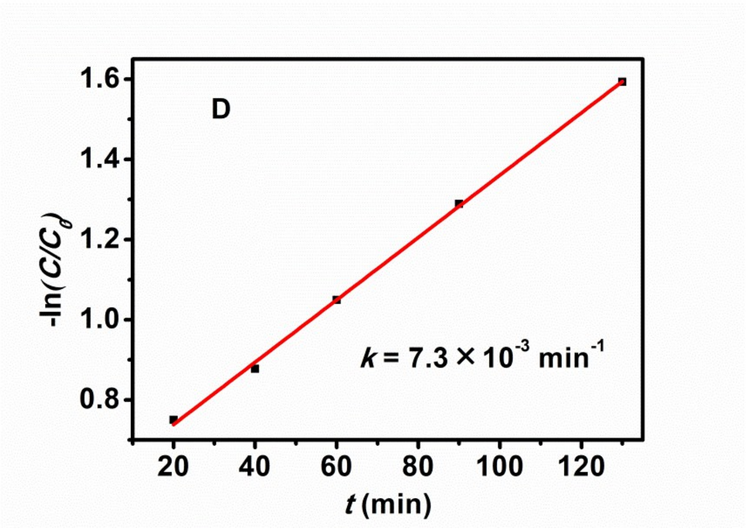

Fig. S8 Rate constant fitting curve of different catalyst supports: (A) ZnO/natural CP, (B) ZnO/CP-150-3, (C) ZnO/CP-150-0.4M5 and (D) ZnO/CP-150-0.4D3.
